# Role of Socio-Psychological Factors in Perceived Quality of Care Rendered by Traditional Medical Practitioners in Ibadan, Nigeria

**DOI:** 10.5539/gjhs.v5n6p186

**Published:** 2013-09-28

**Authors:** Adebayo Adejumo, Moyolebi Faluyi, Adebukola Adejuwon

**Affiliations:** 1 Department of Psychology, University of Ibadan, Ibadan, Nigeria

**Keywords:** traditional medicine, service-quality, influence, socio-psychological factors

## Abstract

**Background:**

It was the aim of the current research to investigate perceived service quality rendered by traditional medical practitioners and the role of socio-psychological factors in the perception.

**Methods:**

The first part, a quantitative cross-sectional survey utilized a 93-item questionnaire to examine the influence of quality of life, general health perception, socio-economic status and personality factors on perceived service quality. The second part, a qualitative study utilized 5 FGDs and 2 KIIs to explore consumers’ evaluation of perceived service quality. Five research questions were raised. The 336 purposively-selected participants were attendees of traditional-health clinics/centers in Ibadan with a mean age of X̄=30.60±9.97.

**Findings:**

The FGD respondents opined that the scope of orthodox-medicine does not cover certain illnesses. 77.8% of the participants attested to the affordability and promptness of services in traditional hospitals; acknowledging that its perceived efficacy (i.e. 56.8%) motivate patronage of traditional-health service. The 2x2x3 ANOVA revealed significant main effect of quality of life (F[1,270]=41.05, p<.001) and socio-economic status (F[2,270]=36.34; p<.001); as well as interaction effect of quality of life, general health and socio-economic status (F[1,270]=9.624, p<.002); while the regression analysis showed independent influence of extraversion (β=0.31; p<.001), agreeableness (β=0.303; p<.001) and openness to experience (β=0.166; p<.01).

**Conclusion:**

This sample acknowledged that traditional health care met quality standards. The role of socio-psychological factors in the quality appraisal was established. The need for better regulation and validation of traditional health care in assuring evidence based care was suggested.

## 1. Introduction

Diseases and threats to health have been, and will continue to endanger human wellness. This forms the background for health care planning and intervention in many societies. Many nations of the world rely on orthodox health systems to meet their health needs. However, too often, and especially in settings of poverty and social inequality, progressive improvement in health care delivery is a rare experience leading to worsening of health disparities. Consciously or otherwise, patients are increasingly appraising the quality of health services vis a vis their expectations, values, and affordability (Igberase, Isah, & Igbekoyi, 2009). Boundaries are disappearing, and the scope of choices for healthcare customers is expanding, giving room to competition.

The president of the Nigeria Medical Association also observed that the public hospital system is characterized by abuse of prescription rights, long-standing on queues (waiting time), and patients’ lack of confidence in the healthcare facilities (Olokor, 2012). For individuals and communities, the latter lead to apathy towards public health services, and considerations of possible alternatives. When the quality, satisfaction, and affordability of available orthodox health service is not assured, coupled with the necessity to maintain personal and family wellness considering available resources and cultural values, there is often a shift of attention to alternative medicine, an example of which is traditional medicine (TM). This may have been in a small measure what prompted the President of Nigeria, Goodluck Jonathan on July 15, 2013 to urge African leaders to “look inwards” and develop “local solutions” to tackle HIV/AIDS and other infectious diseases on the continent (Citizens’ Platform, 2013).

Although the literatures often emphasize orthodox health care or health service based on Western concepts and values, this study in comparison emphasizes the need for greater enquiry into the efficiency and possible satisfaction with culture-driven and tradition-based health care practices in the present day technology-driven health care philosophy. Traditional medicine (TM) involves the use by the folk population primarily of unorthodox and unscientific methods for curative and prevention of diseases (Gyasi, Mensah, Adjei, & Agyemang, 2011). The use of the term traditional should not be interpreted to mean outmoded as TM is assuming greater importance in the primary health care of individuals and communities in many developing countries (Moschik, Mercado, Yoshino, Matsuura, & Watanabe, 2012; Peltzer, Mngqundaniso, & Petros, 2006; WHO, 2002). These approaches to health care belong to the traditions of each culture, and have been handed down from generation to generation (WHO, 1996). Traditional medical practitioners include herbalists, bone-setters, traditional birth attendants, and diviners; and the choice for these services may be influenced by promptness of service, and perceived efficacy of drugs among others (Adaramaja & Baba, 2007).

A review of literature reveals a growing interest of people globally in TM and herbs. About 70% of Australians used at least one form of contemporary and TM, and 44.1% patronized alternative medical practitioners in 2007, with the annual ‘out of pocket’ expenditure on alternative medicine estimated at US$ 3.12 billion (Xue, Zhang, Lin, & Da Costa, 2010). The familiarity of TM practitioners with patients and their communities often provides psychological comfort, especially HIV patients (Gyasi et al., 2011). There are evidences to also show that 60% of the children with high fever due to malaria were successfully treated with herbal medicines in Ghana, Mali, Nigeria and Zambia in 1998 (WHO, 2001). The latter realities urged the World Health Assembly member countries to utilise those medicines (Akerele, 1987) so as to broaden the coverage of health care in their respective countries (Gyasi et al., 2011).

As demands are made for a more patient-centered health care system, it becomes critical to define and measure patient perceptions of health care quality and to understand more fully what drives those perceptions (Sofaer & Firminger, 2005). Perceived Service Quality (PSQ) is defined as the impression that customers have about the overall service provided by the healthcare providers. It is the outcome of an evaluation process where the customers compare their expectations with the service they have received. The patient’s perception of quality of care is critical to understanding the relationship between quality of care and utilisation of health services and is now considered an outcome of healthcare delivery (Reerink & Sauerborn, 1996).

In a Ghana-based study, 58.6% users of traditional health care services claimed that TM services are “cheap” when compared with the orthodox medical services. However, only 7.1% considered the service to be ‘‘expensive’’ relative to the orthodox medical services. TM is therefore the first point of call to many people in the area of study. Different views were given by TM clients in relation to efficacy. 42 (60%) of TM clients and 11 (55%) of the Orthodox Medical Practitioners (OMPs) responded “very good” to the efficacy of TM while 23 clients; 6 OMPs and 5 clients; 3 OMPs respectively responded TM is ‘’good’’ and ‘’bad’’ in terms of efficacy. Some said they have used some types of TM and the effect was positive by achieving the aims for which the medicines were used (Gyasi et al., 2011). The role of gender in the perception of physicians’ cultural competence in health care interactions was investigated by Ahmed and Bates, (2007), no significant difference was observed.

Attitudinal and socio-behavioural factors often underlie health care decisions on the part of both the provider and seeker of health service (Gray, 2007). The health care utilisation of a population is related to the availability, quality and cost of services, as well as to social-economic structure, and personal characteristics of the users (Manzoor, Hashmi, & Mukhtar, 2009; Onah, Ikeako, & Iloabachie, 2009). Exploring these concerns will inform decision towards its improvement and sustainability (Gyasi et al., 2011). Although Oyebola (1980) and Olutayo (1993) observed that even in such places like Ibadan where modern medical facilities exist, a large percentage of the patients still visit traditional healthcare services. Despite the latter, individual perceptions of the role of TM is not explicitly studied in Nigeria.

The psychosocial variables of interest in this study are Quality of Life (QoL), General Health (GH) perception, and personality and socio-economic status (SES). Perceived QoL can be defined as the level of wholesomeness (or otherwise) observable in the degree of happiness that an individual derives from life and health (Patrick & Marion, 2008). Perceived GH rating seeks to understand an individual’s comprehensive definition of personal health status. An individual’s personality is the combination of characteristics or qualities that form the individual’s distinctive character. In psychology, the Big Five personality traits are the five domains that are used to describe the human personality in general and are: conscientiousness, agreeableness, neuroticism, openness to experience and extroversion. Openness is a concept referring to the degree of intellectual curiosity, creativity and a preference for novelty and variety a person has; Conscientiousness is about a tendency to show self-discipline, act dutifully, and aim for achievement; Extraversion is associated with positive emotions, surgency, assertiveness and sociability; Agreeableness means a tendency to be compassionate and cooperative rather than suspicious and antagonistic towards others; while neuroticism describes an individual’s tendency to experience unpleasant emotions easily, such as anger, anxiety, depression or vulnerability (R. L. Atkinson, R. C. Atkinson, Smith, Bem & Nolen-Hoeksema, 2000). Socioeconomic status (SES) is an economic and sociological combined total measure of a person’s work experience, available resources and rank in the society.

In this study we investigated consumers’ perceived quality of care rendered by TM practitioners in Ibadan, Nigeria from the customers’ perspective, and the role of psychological and social factors in the perception. Attempts were also made to answer the following questions:

- What motivates the choice of TM service, and how do consumers perceive the quality of health care provided in traditional health care institutions?
- Is there any main and interactive effects of QoL, GH perception and SES on PSQ among consumers of traditional health care?
- What is the role of personality factors in the perception of the quality of care among consumers of traditional health care?
- Is there any significant effect of gender on the PSQ among consumers of traditional health care?

## 2. Method

### 2.1 Design

The study consisted of two phases. The quantitative part was a survey which employed a cross sectional design to examine the influence of QoL, GH perception, SES and personality factors (conscientiousness, agreeableness, neuroticism, openness to experience and extroversion) on PSQ among consumers of traditional healthcare services. The independent variables were considered at two levels each except SES at three levels.

In order to have comprehensive information to describe the context, or natural setting, with a view to having a broader understanding of the entire situation, a qualitative study was conducted. It was based on grounded theory (Charmaz, 2006) and included five Focus Group Discussions (FGDs), and two Key Informant Interviews (KIIs) aimed at seeking knowledge about what motivates patronage of traditional health centres, and the consumers’ evaluation of the PSQ.

### 2.2 Setting

The study took place in Ibadan, the capital city of Oyo state, Nigeria and also the largest city in West Africa. The setting offers the advantage of availability of both orthodox and traditional health services, with each provider claiming superiority. The first teaching hospital (University College Hospital) in Nigeria and a variety of registered traditional health care service providers are located in this former regional capital.

### 2.3 Participants

The participants comprised of 336 purposively selected respondents patronizing three prominent traditional health care centers in the city. Two hundred and twenty (65.5%) were males while 116 (34.5%) were females. Two hundred and three (60.4%) were Christians, 127(37.8%) were Muslims while 6 (1.8%) adhered to other faiths/beliefs. Their age ranged between 25 and 75 years with a mean of (*X̄*=30.60±9.97. Their educational background also varied; 178 (53%) obtained a polytechnic/university degree, 67 (19.9%) had National Diploma or equivalent certificate, 32 (9.5 %) had no formal education, 26 (7.7%) completed junior school education, 21 (6.3%) completed primary school education, 12 (3.6%) completed secondary school education. Concerning their monthly earnings, 98 (26.7%) did not disclose their monthly earnings, 60 (16.4%) earned between N60,000 – N99,000, 55(15%) earned between N20,000 – N59,000 a month, 52 (14.2%) earned between N100,000 – N149,000, 41(11.2%) earned above N400,000 a month, 32(8.7%) earned below N20,000 a month, while 28 (7.7%) earned between N200,000 – N399,000, where one United States dollar exchanges for approximately N157. Concerning participants’ self-rating of their SES, 112 (33.3%) reported being in the medium SES, 87(25.9%) reported being in the low SES, 82(24.4%) reported being in the high SES, while 55(16.4%) did not disclose their SES.

### 2.4 Pilot Study

A pilot study was conducted to pre-test the research instrument, and also to determine the feasibility of the main study. This was followed by submission of the research protocol for ethical approval, which was granted by the ethics committee.

### 2.5 Instruments

The survey part of the study adopted the use of a 93–item structured self–report questionnaire which was divided into 5 sections: The 14-item Section A was designed to obtain participants’ socio-demographic information including gender, age, marital status, and religion among others. Seven of the items were designed to measure the quality of care received in a traditional health institution.

Section B consisted of the 19-item Perceived QoL scale developed by Patrick and Marion (2008). The perceived QoL scale contains items testing perceptions of one’s position in life in the context of the culture and value systems in which one lives. It has an 11-point response scale where a mean of <7.5 means dissatisfied, and vice versa. It has a correlation coefficient of 0.70. A revalidation of the scale yielded an alpha co-efficient of 0.83.

Section C of the questionnaire consisted of the 28-item General Health Questionnaire (GHQ28) developed by Goldberg and Williams (1988) to assess four general aspects of wellness of an individual. Responses are scaled from 0 to 4, with a maximum score of 84. High score means poor perceived GH. Reliability coefficients have ranged from 0.78 to 0.95 in previous studies, while the total possible score on the GHQ 28 ranges from 0 to 84 (Ahumada & Alvarez, 2004).

Section D contained the 10-item Big-Five Personality Inventory (BFI) developed by Rammstedt and John (2007). The reliability coefficients as reported by the authors are; extraversion=.87, agreeableness=.74, conscientiousness=.84, neuroticism=.88, and openness to experience =.79. The higher the score above the global mean score on each subscale, the higher the individual’s trait on that particular personality factor, and vice – versa.

Section E of the questionnaire consisted of the 22-item PSQ SERVQUAL scale developed by Parasuraman, Zeithaml and Berry (1988). Responses are scaled from 1 to 7, ranging from strongly agreed to strongly disagree. The higher (less negative) the score, the higher the level of PSQ. Its reliability ranges between .87 and .90, with a mean score of (*X̄*=70.57±10.01.

For the qualitative study, a 4-item FGD guide questions was used to seek participants’ opinions regarding questions which included:

- In your opinion, why do members of this community seek the services of traditional health care service providers?
- How would you rate the quality and outcome of service/care rendered by workers in traditional health care centers in Ibadan?

### 2.6 Procedure

The ‘guide’ questions for the qualitative study, as well as the research questionnaire were interpreted into Yoruba, the local language. A pilot study was conducted, followed by ethical approval. The researchers also sought the permission of the head of the traditional health centers. Thereafter, people visiting TM practitioners in each of the health centers were approached; the purpose, risks and benefits of the study were explained. Potential participants were assured of their confidentiality, and that their treatment would not be tied to their decision to participate in the study, or otherwise. Informed consent processes were duly followed.

Willing participants were given either the English or Yoruba version of the questionnaires as appropriate. The questionnaire took an average of 27 minutes to complete, some however held on to it till the next day. Of the 395 questionnaires administered, only 334 were correctly completed and returned, representing 84.6% response rate. The returned questionnaires considered adequate for data analysis were coded, stored and entered for data analysis using the SPSS 19 version of computer software package. Following the administration of the questionnaires, participants willing to be part of the qualitative study were enrolled appropriately.

During each qualitative survey session, there was a moderator and note taker. All interviews were recorded with the permission of the participants. After the interviews, the recordings were transcribed into computer files. Care was taken to assure the respondents that any possible identifier would be removed from any subsequent report. As soon as the final research report was written, the tapes from the interviews were destroyed.

Five FGDs were conducted in three traditional hospitals. The first was in a traditional bone setting hospital where a combination of 5 male and 3 female (4 Christians and 4 Moslems) motorcycle accident victims (with fractures) receiving traditional bone setting were involved in group discussion; the second was in a naturalist hospital where two FGDs (one each for males and females) were conducted; the third setting was in a Herbal Centre where two FGDs were held; one for Yoruba speakers, and the other for people willing to communicate in English language. A high school teacher and a nursing mother who came for consultation and treatment by the traditional doctor in the naturalist hospital were engaged in separate IDIs one after the other.

### 2.7 Data Analysis

The data analysis consisted of two parts. The first part involved the management of the data generated from the qualitative study. All of the discussion and interview transcripts were read by the researchers and coded in the style of grounded theory approach to data analysis (Charmaz, 2006). Three category headings were generated from the data and under these all of the data were accounted for. Two independent researchers were asked to verify the seeming accuracy of the category system and after thorough review, minor modifications were made.

Analysis of the data for the quantitative study included descriptive statistics such as mean and standard deviation, as well as inferential statistics such as multiple regression, and 2x2x3 ANOVA at p<0.05.

## 3. Results

The findings from the analysis of the qualitative data, as well as the result of the analysis of the quantitative data are hereby presented.

### 3.1 Findings

Presentation of findings from the qualitative study in line with the main themes and research questions are presented below:

#### 3.1.1 The first research question sought to explore the motivation for patronizing traditional medical practitioners. The analysis of the participants’ responses is hereby presented:

A number of respondents found that long before the advent of orthodox health practice, the fore fathers of Yorubas had unique ways of treating diseases. Many ailments can only be treated by the *adahunses*, and the *oniseguns*. The former are more powerful in handling physical, mental and some spiritual disorders, using both local medications and some mystical powers e.g. incantations; whereas the *oniseguns* rely on herbs and medicinal products handed over from generation to generations in managing health concerns of the people. For most of the respondents, these cannot be assured in Western medical practice. These viewpoints were however varied based on participants’ peculiar socio-psychological characteristics.

One FGD participant expressed in the local language that: *“Ki agbado to d’aye, nkan kan saa ni adiye nje. Olaju lo le wa de idi ogun oyibo. Tenin’teni, egboogi ibile ni won fi bi wa, oun lo si ye ki a gbajumo..”* meaning that before the advent of western medicine, our people had survived on local medicines. It is globalization that drew our attention to orthodox health care, many of us were raised with use of local herbs, and so we should focus on it.

In the opinion of a participant, “some members of the public desire traditional health care because of its perceived strength, affordability, and accessibility. These also motivate some people to patronize patent medicine vendors. Good doctors and nurses keep leaving our government hospitals; where else do we go if not to trust the native herbs.’’

#### 3.1.2 The second research question sought to discover participants’ evaluation of the quality and outcome of healthcare service received from TM service providers. The analysis of the participants’ responses is hereby presented:

Seven of the nine (77.8%) of the participants in the Yoruba FGD were of the opinion that apart from affordability, there is a greater level of privacy, human relations and individualized attention in traditional health care centers. The two interviewees in the KIIs also unanimously expressed that no matter its flaws, some people will prefer traditional health service because there are many disorders in the society that western medicine does not cure easily.

A school teacher participant said:

“Look at the problem of HIV for instance, so much of human and financial resources have been squandered on its control, but where are we today? The disease has killed so many over the years, yet if half of that attention had been given to our native doctors, they would have curtailed the virus by now.”

A motorcycle accident victim with fractured fibular retorted

“I was taken to Universal Teaching Hospital (correct name withheld) after the accident. For two days, it was one test or the other, with so many bills pilling up, yet nothing concrete was achieved. But see now (pointing to the local sticks and herbs rubbed on the fracture site), within a week the pain is reducing, and baba i.e. the attending physician has assured me that I will go back to my commercial motorcycling job within the next 8 weeks, compared to the three months the other medical doctor was saying earlier.”

There were dissenting views among study participants at the Herbal Centre. While 4(50%) of the 8 FGD participants felt that traditional treatment could match orthodox treatment in many perspectives, they however observed that it could be less reliable in the sense that its regulation and control mechanisms are weak at the moment. One participant was quoted as saying that

“Current practices in the form of converting these herbs to caplets, and improved packaging is commendable. But it is only God that can make one identify which of them is fake or genuine. The current level of unemployment, corruption, and quackery in this country could make some people to claim to be competent, without people really knowing that they are being exploited. Additionally, many of the traditional doctors today learnt in part. The totality of the original traditional treatment modalities were not fully handed over to the present generation. This might have accounted for the drop in standards nowadays. Standards are falling everywhere despite the so called modernization. However, just like in Teaching Hospitals, those who are good are simply good.”

Three (37.5%) rather felt that traditional health offers better outcome in that it is broader in scope. One participant said

“Traditional medical care is better in managing some problems that may not be due to microorganisms or clear causative pathways, such as asasi or eedi (i.e. bewitchment or demonic attack). For example, one may be sick without any clear answers to how and why. It would be disastrous to stick to medical doctors in such situations; whereas a sound native doctor would know how to investigate and manage it better.”

### 3.2 Results

Presentation of results from the quantitative study in line with the research questions are presented below:

In order to find out participants’ quantitative evaluation of the PSQ among TM practitioners, their responses were analyzed and presented in a descriptive statistics Table below.

Table 1 showed that of the 2352 maximum possible responses, 1335 i.e. 56.8 % of the participants’ responses supported the view that the quality of health care received TM practitioners met their standards of quality; while 31.7% disagreed, and 11.5% either reported that they do not know or abstained from responding.

**Table 1:** Descriptive statistics Table showing participants’ evaluation of the quality of health care received from traditional medical practitioners

S/N	Criterion of quality	Agreed		Disagree		Don’t know	

		N (N=336)	%	N (N=336)	%	N (N=336)	%
1	History taking	218	64.9	107	31.8	11	03.3
2	Quality of Investigation	147	43.8	132	39.2	57	17.0
3	Privacy and confidentiality	198	58.9	96	28.6	42	12.5
4	Promptness and attention	229	68.2	91	27.0	16	04.8
5	Adequacy of drugs & Treatment	179	53.3	104	30.9	53	15.8
6	Affordability	161	47.9	143	42.6	32	09.5
7	Accessibility	203	60.4	73	21.8	60	17.8
	Average total	1335/2352	56.8	746/2352	31.7	271/2352	11.5

iii. The third research question focused on whether there will be main and interactive effects of QOL, GH perception and SES on PSQ. It was tested with 2x2x3 ANOVA.

Table 2 showed that there was significant main effect of QoL [F(1,270)=41.05, p<.001] and SES [F(2,270)=36.34; p<.001] on PSQ, while GH did not [F(1,270)=0.167; p>.05]. QoL and SES [F(2,270)=52.55; p<.001], as well as GH and SES had significant interaction effects on PSQ [F(2,270)=17.839; p<.001], while QoL and GH did not [F(1,270)=3.70; p>.05]. Based on these significant interaction effects, a post-hoc test was done for multiple comparisons.

**Table 2:** 2x2x3 ANOVA of main and interactive effects of QOL, GH and SES on PSQ

Source	SS	Df	M	F	Sig.
Quality of Life (A)	3263.219	1	3263.219	41.045	.000
General Health (B)	13.269	1	13.269	.167	.683
Socio-Economic Status (C)	5778.328	2	2889.164	36.340	.000
A * B	294.200	1	294.200	3.700	.055
A * C	8355.816	2	4177.908	52.550	.000
B * C	2836.458	2	1418.229	17.839	.000
A * B * C	765.120	1	765.120	9.624	.002
Error	21465.857	270	79.503		
Total	47941.580	280			

Table 3 showed that participants with high QoL and medium SES reported significantly higher PSQ (*X̄*=59.27) than those with low QoL and low SES (*X̄*=32.23) with mean difference of 27.04. Also, participants with high QoL and medium SES reported significantly higher PSQ (*X̄*=59.27) than those with low QoL and high SES (*X̄*=47.37) with mean difference of 11.90. Furthermore, participants with high QoL and medium SES reported significantly higher perceived PSQ (*X̄*=59.27) than those with low QoL and medium SES (*X̄*=51.44) with mean difference of 7.83.

**Table 3:** LSD multiple comparison test showing effects of QoL, SES and GH PSQ

Quality of life	SES	N	*X̄*	SD	1	2	3	4	5	6

1. Low	High	41	47.37	12.36	-					
2. Low	Medium	34	51.44	8.65	-4.07	-				
3. Low	Low	39	32.23	11.80	15.14*	19.21*	-			
4. High	High	41	44.59	14.71	2.78	6.85*	-12.36*	-		
5. High	Medium	78	59.27	5.80	-11.90*	-7.83*	-27.04*	-14.68*	-	
6. High	Low	48	56.72	3.92	-9.35*	-5.28*	-24.49*	-12.13*	2.55	-

General health	SES	N	*X̄*	SD	1	2	3	4	5	6

1. Low	High	35	40.00	13.81	-					
2. Low	Medium	52	57.02	9.37	-17.02*	-				
3. Low	Low	80	45.10	15.22	-5.10*	11.92*	-			
4. High	High	47	50.43	11.66	-10.43*	6.59*	-5.33*	-		
5. High	Medium	60	56.47	5.73	-16.47*	0.55	-11.37*	6.03*	-	
6. High	Low	7	50.00	0.00	-10.00*	7.02*	-4.90	0.43	6.47*	-

* *P* <.05

It could also be seen that participants with low GH and medium SES reported significantly higher PSQ (*X̄*=57.02) than those with low GH and high SES (*X̄*=40.00) with mean difference of 17.02. Participants with high GH and medium SES reported significantly higher PSQ (*X̄*=57.02) than those with low GH and low SES (*X̄*=45.10) with mean difference of 11.92. Moreover, participants with high GH and medium SES reported significantly higher PSQ (*X̄*=56.47) than those with low GH and low SES (*X̄*=45.10) with mean difference of 11.37.

iv. The fourth research question sought to know whether personality factors will have significant independent and joint influences on PSQ. It was tested with multiple regression.

Table 4 showed that personality factors jointly predicted PSQ (F(5,330)=37.924; R^2^=0.365; p<.001), accounting for 37% of the variance of PSQ, while the remaining 63% could be due to the effect of extraneous variables. Therefore, the prediction of PSQ by the predictor variables was not due to chance.

**Table 4:** Multiple regression analysis showing the big-5 personality traits’ prediction of PSQ

Variables	B	SE	P	T	P	F	R^2^	P
Extraversion	2.271	0.347	0.310	6.540	<.001			
Agreeableness	2.574	0.424	0.303	6.064	<.001			
Conscientiousness	0.320	0.421	0.039	0.780	>.05	37.924	0.365	<.001
Neuroticism	0.128	0.406	0.019	0.317	>.05			
Openness	1.199	0.405	0.166	2.960	<.01			

Furthermore, extraversion (β=0.31; p<.001); agreeableness (β=0.303; p<.001); and openness to experience (β=0.166; p<.01) had significant independent influence on PSQ, meaning that these factors that could influence an individual to perceive the services of traditional medical practitioners as good. However, conscientiousness (β=0.039; p>.05) and neuroticism (β=0.019; p>.05) did not independently predict PSQ.

v. The fifth research question sought to find out whether female participants will score significantly higher than males on PSQ. It was tested with the use of independent sample t-test.

Table 5 revealed that there was no significant effect of gender on PSQ (t=0.056; df=334; p>.05) as male participants did not significantly report higher PSQ (*X̄*=50.25) than females (*X̄*=50.16).

**Table 5:** T-test analysis comparison of gender on PSQ offered by TM care providers

Variables	Gender	N	*X̄*	SD	Df	t	P
Perceived quality of healthcare service	Male	220	50.25	12.75			
	Female	116	50.16	13.62	334	0.056	<.05

## 4. Discussion

This study was designed to investigate participants’ evaluation of the perceived quality of service rendered by TM practitioners, as well as the role of psychological and sociological factors affecting the evaluation. The respondents were of the opinion that the cause of some ailments cannot be explained through scientific physiological pathways; it would therefore be inappropriate to seek treatments for such conditions through orthodox health service. Many participants were motivated to utilize TM because of the belief that it is stronger, more accessible and affordable compared to orthodox treatment. Privacy of patients, better human relations, and more individualized attention among others offered by TM service providers primarily motivated patronage of the service. However, the issue of regulation of TM service was perceived as weak. More than half of the participants acknowledged that TM service providers met their standards of quality.

The quantitative analysis revealed a significant main effect of QoL and SES on PSQ, while GH did not. QoL and GH had no significant interaction effect; while QoL, GH and SES yielded additional significant joint influence on PSQ. Personality factors jointly predicted PSQ. Extraversion, agreeableness and openness to experience also had significant independent influence, while no gender difference was established in the evaluation of the PSQ. Overall, the results of this study established the acceptance and utilization of TM services, suggesting the need not only for a greater attention to its improvement, but consideration of the psychological and socio-demographic factors that determine its appreciation.

The finding suggests that factors such as; belief in tradition, more detailed history taking, promptness in attention, and accessibility motivated the respondents in the qualitative study to seek TM services attest to the priority placed on the variables by the sample. This supports in part the outcome of some earlier investigations (Gyasi et al., 2011; Adaramaja & Baba, 2007), but reveals the relevance of additional factors as reported by the participants. Another possible explanation for this is that TM service provision has to do with culture, and culture is dynamic hence the variability of the cultural determinants motivating the choice of TM services in diverse settings. Further, cultural factors could also influence the perceived pathophysiology, severity, and vulnerability to a disease.

Concerning the PSQ and efficacy of TM services, there were variations in the context and content of the participants’ evaluation. This was exemplified in the 77.8% to 37.5% of the qualitative study participants’ approval of TM services, with a fairly similar percentage of the participants in the quantitative study supporting the view that the PSQ rendered by TM service providers met their standards of quality. This buttresses the observation by Peltzer, Mngqundaniso and Petros (2006) that TM is assuming greater importance in the primary health care of individuals and communities in many developing countries, such that the President of Nigeria could recommend same to other African leaders brainstorming on how to meet the health challenges of the African continent as shortly as possible (Citizens’ Platform, 2013).

This study revealed significant main effect of QoL and SES on perceived quality of healthcare, while GH did not. Significant interaction effect of QoL and SES, as well as that of QoL, GH and SES were also discovered. This could be interpreted or hypothesized to mean that in comparing the means generated by the three variables, GH is insignificant. Before now, there is little knowledge of the possible effect of QoL and SES on PSQ, especially among consumers of TM service. This suggests that an individual’s level of happiness derived from life and health, as well the level of combined measure of his economic and social rank could influence the individual’s PSQ. This becomes more meaningful when considering the following two reasons. Firstly, despite the oil wealth available in Nigeria, close to seventy percent of the population still lacks any form of health insurance. Secondly, these factors also relate to possession of human and material resources, which influence accessibility and affordability of social services; including health care. This supports the evidence presented by the WHO that as many as 60% of children in developing countries including Nigeria opted for and get treated with herbal medicine (WHO, 2001), whereas the children of the fewer elites would likely have access and be offered expensive “state of the art” orthodox health care (Manzoor et al., 2009; Onah et al., 2009) that are often within the reach of a few, especially the political class.

The association between personality factors and PSQ lend support to the relevance of individuals’ unique and distinct characteristics in choices and preferences. People with openness for experience desire invention, adventure, curiosity, and variety of experience. People with agreeable character are often compassionate and cooperative, while an extraverted person is assertive, sociable, and has the tendency to seek stimulation in the company of others (Atkinson et al., 2000). It implies that there is a relationship between these traits and people’s evaluation of PSQ, suggesting the need for considering such traits in predicting evaluation of quality of health care.

No gender differences were established in the evaluation of PSQ received from TM service providers. This supports the findings of Ahmed and Bates (2007) among respondents in three Ohio, USA counties. A possible explanation why gender difference was not supported by the data might be that gender is a variable inherent in people’s culture, and associated with perceptions of quality of service and satisfaction in health care interactions. It implies that in the context of health care provider-patient interaction in Ibadan, gender may become a ‘non-significant’ variable in evaluating PSQ rendered by TM service providers. Despite this, there might exist Nigeria in-country differences because of the gross diversity in the culture and social characteristics of the people.

### 4.1 Limitations

This study is not without limitations. Many of the participants seek TM care because of the privacy, personalised service and promptness which they claim could hardly ever be assured in public teaching hospitals. Contrary to their expectations, researchers still come over to seek their enrolment in research related activities which betrays the confidentiality and privacy expected in traditional care settings. This might have affected the veracity of their responses. Additionally, the adoption of both qualitative and quantitative data collection techniques generated a massive data set beyond the contents of the results presented in this paper. However, these provided a good opportunity for not only understanding the content and context of the participants’ responses but also allow for a comparison of both data gathering methods, which in this study is highly similar, suggesting that the results are reliable.

## 5. Conclusion

This study established that despite the diversity in the opinion of the respondents, the larger majority of TM service customers acknowledged its quality in interviews and survey questionnaire responses. This provides a vital clue towards resolving the current controversy on the efficacy and acceptability of TM in this society. The role of QoL, SES, openness for experience, agreeable character, and extraversion personality traits in the evaluation of PSQ was also established. However, it is likely that the current increasing demand for TM care in Ibadan and similar settings will require regulation and standardisation, or at least further effort to scientifically validate its processes and outcome. Without these, the consumers of TM services will be denied evidence based care; their cultural beliefs and socio-economic status not-withstanding.
